# Dr. Stevenson, on Vaccine

**Published:** 1802-01

**Authors:** John Stevenson

**Affiliations:** Kegworth


GENTLEMEN,

AN ingenious Correſpondent requeſts, through the medium of your Journal for October laſt, to be informed, "Whether the virus of the Cow-Pox, taken after the formation of the areola, never produces the genuine diſeaſe? or, whether it only ſometimes fails?" To this enquiry you reply, in a Note, that "though you believe it will not always fail, yet that it is not to be relied on as a preventive of the Small-pox, even though it aſſume ſuch a ſtrong reſemblance to the genuine as not to be *diſtinguiſhed by any deſcription.*"

Encouraged by the attention you have paid to the above queſtion, may I be permitted to ſolicit your *reaſons* for denominating *my* Caſes of Cow-Pox, as related in a former Number of your very intereſting Journal, *ſpurious?* This appellation decidedly militates againſt the whole ſcope and ſpirit of the Communication, which is intended to repreſent them as *examples* of failure in the preventive influence of the *genuine* Vacciola. I was tempted to lay them before the public from the belief, that in this, as in other diſeaſes, where no *deſcribable difference* appeared in the phenomena, the *genuine* and *ſpecific*, and not the *ſpurious* or *imitative* ſpecies, exiſted. As, however, from the appellation affixed to the hiſtories alluded to, (Mr. S. on the *ſpurious* Cow-Pox) it ſeems that you were impreſſed with different ſentiments, allow me to aſk, as a counterpart to your Correſpondent's propoſition, Whether a *ſpurious* Cow-Pox can be occaſionally communicated by Vaccine virus taken *prior* to the appearance of the efflorefcence, as is acknowledged to be the caſe with matter procured *after* that period? And does the ſame *indeſcribable difference* obtain in reſpect to its ſymptoms, and is it equally fallacious with the imitative ſpecies produced by virus taken after the formation of the areola?

If theſe poſitions can be experimentally and unequivocally anſwered in the affirmative, my Caſes muſt be deemed *ſpurious*; and, as ſuch, I ſhall ſubſcribe to the propriety of their preſent deſignation, and to my own miſconception of their real character. I feel much intereſted in the ſolution of theſe queries, from having read in an extract from a little production on the ſubject of Cow-Pox, (lately publiſhed by the elegant and poetic pen of Dr. Lettſome) a direct aſſertion to the contrary.

I cannot diſmiſs this Paper without making it the vehicle of ſome curſory obſervations on the vague uſe of the epithet *ſpu-*

*rious* or *imitative*, as expreffive of a deceptive fpecies of Cow-Pox, and on the abfolute want of its diagnofis.

"It is a great abufe of language," fays Mr. Locke, "to make ufe of words to which we have no fixed or determinate idea." I am apprehenfive that if we examine fcrupuloufly into the technical import of the terms fpurious* and imitative, we fhall find caufe to fufpect their mifapplication in a critical and nofological fenfe to the Vacciola; for what do thefe words imply, when ufed on other medical fubjects? Do they not convey the idea of great *similarity*, rather than an *indescribable difference* in the features of a difeafe? Thus, *e. g.* we fay that the Chicken-Pox is imitative of the Cow-Pox; the Rheumatifm of the Gout, &c. Again, we annex the adjective *spurious* to a certain fpecies of Pleurify. But in thefe inftances the words have a fixed and determinate fignification, becaufe all of them difplay fome difcriminative fymptoms, which will enable an attentive practitioner to diftinguifh each refpective difeafe. What characteriftics does the Cow-Pox furnifh, to lead to a conviction of its exiftence? On this point we may fay with Horace, "Decipimur fpeciê recti." To call that an imitative or fpurious complaint, which exhibits a concourfe of phenomena fo truly indicative of the genuine, as not to be *distinguifhed by any description*, is certainly a contradiction in terms, a diftinction without a difference; at leaft, the fimilarity between the fpurious and genuine Vacciola is fo clofe and intimate, that, like the lineaments of the Sifters in Ovid, a more than ordinary fhare of refinement in the art of phyfiognomy and nofological difcrimination is requifite, in order to recognize the one from the other.

"——— Facies non omnibus una,

"Nec diversa tamen."

Failures, I believe, are by the abettors of Vaccination, univerfally maintained to be the *poffible* refult *only* of the *spurious* fort. Shall we then, notwithftanding this momentous, and, I truft, not forced declaration, lay upon our oars, fufpending our further refearches, and beftow our unqualified eulogium on this grand innovation, and yet tacitly acknowledge that we are not at

* The Editors are convinced that they have fometimes ufed the term *spurious* in a too general, and therefore in an incorrect manner.

There is a *spurious* puftule which can be propagated by inoculation; but when the well conducted, or at leaft well intended, endeavours of a careful practitioner, fail to produce genuine vacciola, the difeafe fo produced fhould be called *insufficient*, *imperfect*, *defective*, *&c.* in the firft inftance; and *spurious* only when the matter has been *taken* from a fpurious or imperfect puftule. This fubject will be refumed in a future Number.

at prefent in the poffeffion of accurate criteria whereby to decide on its actual prefence?

I fhall beg leave to conclude thefe defultory remarks with the two following inferences.

1ft. It appears from the above ftatement, that the terms *spurious* and *imitative* are ufed in a very loofe, indefinite fenfe.

2dly, and principally. That a correct *definition* of the *spurious* Vacciola, expreffive of the fymptoms or appearances by which it is fpecifically diftinguifhed from the *genuine*, is ftill a *great desideratum*; fince, by the want of it, the practitioner is placed in a fomewhat fimilar fituation to that of a mariner at fea without his compafs.

I am, &c.

*Kegworth, Nov.* 17, 1801. JOHN STEVENSON.

---

